# Construction of a Joint Prediction Model for the Occurrence of Ischemic Stroke and Acute Myocardial Infarction Based on Bioinformatic Analysis

**DOI:** 10.1155/2022/5967131

**Published:** 2022-04-04

**Authors:** Zhaolei Ma, Lan Chu, Chun-Feng Liu, Wupeng Liu, Jing Wei

**Affiliations:** ^1^Department of Neurology and Clinical Research Center of Neurological Disease, The Second Affiliated Hospital of Soochow University, Suzhou, Jiangsu 215004, China; ^2^Department of Neurology, The Affiliated Hospital of Guizhou Medical University, Guiyang, Guizhou 550004, China; ^3^Institute of Neuroscience, Soochow University, Suzhou, Jiangsu 215004, China; ^4^Department of Cardiology, The Affiliated Hospital of Guizhou Medical University, Guiyang, Guizhou 550004, China; ^5^Department of Endocrinology, The Affiliated Hospital of Guizhou Medical University, Guiyang, Guizhou 550004, China

## Abstract

Ischemic stroke (IS) has imposed significant threat to both middle-aged and elderly people worldwide. Acute myocardial infarction (AMI) is a rare but serious complication following IS, which can further increase patient disability and mortality rates. With the development of intravenous thrombolysis and endovascular treatment, the prognosis of IS has been greatly improved. However, the pathogenesis of IS complicated with AMI is still unclear. To fill this gap, this work uses bioinformatic analysis, where IS and AMI datasets were combined for differential gene analysis, and then, a ROC prediction model for target gene analysis was constructed. It is found that OSM gene has the highest prediction accuracy (AUC = 0.793), followed by IL6ST, IL6, JAK1, IL6R, and JAK2 genes. Joint prediction model showed higher accuracy in predicting the outcome of control and case (AUC = 0.918). The etiology of ischemic stroke and acute myocardial infarction is complicated. Their cooccurring pathological mechanisms and the conversion between the two diseases could not be explained by a single gene. Therefore, the joint prediction model in this work can provide a better prediction accuracy for research purpose.

## 1. Introduction

Stroke, ischemic heart disease, and malignant tumors are currently the three leading causes of death worldwide, among which, stroke, the second leading cause of death, poses a serious threat to human health. According to statistics, about 5.5 million people die of stroke every year globally. Without timely treatment since the onset of stroke, various degrees of physical and mental disabilities may be caused, which will lead to a great burden on individuals, families, and the society. Based on the findings of a national disease burden study, stroke has become the first cause of death in China [1]. The overall prevalence, annual morbidity, and mortality in China are 11,148 per million, 2,468 per million, and 1,148 per million, respectively [2]. 70% of stroke patients are of the ischemic stroke (IS) type, and 20% are of the cardiogenic stroke (CES) type. Chinese epidemiological survey has found that the 1-month mortality rate of acute IS patient is about 2.3%-3.2%. The 3-month mortality rate is 9%-9.6%, with a fatality or disability rate of 34.5%-37.1%. The 1-year mortality rate is about 14.4%-15.4%, with a fatality or disability rate of 33.5%-33.8% [3]. For CES, atrial fibrillation (AF) is the most important risk factor, which contributes to 80% of the CES patients. Worse prognosis of IS patients with AF has been reported [2, 4, 5].

Studies have shown that acute myocardial infarction (AMI) is closely related to the occurrence of stroke. About 80% of AMI patients have IS within a year, of which 80% occur within 2 weeks after AMI [6]. New-onset or chronic AF and histories of past stroke are important predictors of IS after the occurrence of AMI [7]. A meta-analysis of 47,229 patients involving 8 studies showed that even for patients with acute IS but without any heart disease history, the AMI incidence within 1 year of stroke is still up to 3% [8]. Meanwhile, 1/3 of the acute IS patients have asymptomatic coronary artery stenosis (stenosis ≥ 50%) [9]. Surveys on the risk factors of IS and haemorrhagic stroke in 22 countries show that the 10 risk factors including AMI can account for 91.5% of the population attributable risk in IS patients globally [9].

The current limited epidemiological data shows that the heart and brain are closely related, since they have a mutual hemodynamic and pathophysiological basis. However, due to their anatomical differences, it is difficult to determine either the heart- or brain-originated factors are the leading causes. This article aims to use bioinformatic analysis to predict the target genes of IS and AMI, which could indicate the possibility of AMI occurrence when IS occurs and vice versa, so one can achieve early prevention of either disease.

## 2. Materials and Methods

### 2.1. Data Collection

Target chip was searched and downloaded through GEOquery package from the Gene Expression Omnibus (GEO) chip database [10]. Briefly, the following datasets were downloaded: (1) IS dataset (GSE22255) consisted of 20 normal control groups and 20 IS groups. (2) CES dataset (GSE58294) consisted of stroke-derived stroke contains 23 normal control groups and 69 patients in the cardiogenic stroke group. (3) AF combined with stroke (AF/IS) dataset (GSE66724) consisted of 8 cases of AF combined with stroke group and 8 cases of simple AF group. (4) AMI dataset (GSE66360) consisted of 49 cases of AMI patient group and 50 normal control groups. Probes that corresponded to multiple molecules were removed. For multiple probes that corresponded to the same molecule, only the one with the highest signal value was used. ComBat function from the sva package was used to remove the interbatch difference for the above filtered datasets (different datasets are regarded as the interbatch difference). The box plot was used to assess the outcome of data normalization; principal component analysis (PCA) plot was used to assess the clustering situation between groups; limma package [11] was performed to study the differential expression between the two groups. ∣Log2FC | ≥1 and adjusted *p* < 0.05 were used as the cut-off criterion for differential gene expression analysis. A negative number represent downregulation, and a positive number represent upregulation. By applying the above criteria, DEGs were determined by comparing the normal control group and the stroke group, the normal control group and the myocardial infarction group, the myocardial infarction group and the myocardial infarction with stroke, and the myocardial infarction with AF group, respectively. Probes are then converted into gene names through the GPL570 platform. ComplexHeatmap package was used to generate heat maps [12], and R language ggplot2 [12] package was used to generate the volcano maps. Online Venn analysis tool (http://bioinformatics.psb.ugent.be)/webtools/Venn/) was used to obtain the intersection genes of IS, CES, AMI, and AF, respectively. The R language ggplot2 package Venn diagram was used to visualize these intersecting genes for subsequent analysis.

### 2.2. Tissue-Specific Gene Expression

Tissue-specific expression of DEGs was analysed by using the online resource BioGPS (http://biogps.org). Transcripts mapped to a single tissue that meet the following criteria were identified as having high tissue specificity: (1) tissue-specific expression levels were 10 times higher than the median value of other tissue, and (2) the highest expression level for the second-highest expression was no higher than 1/3 of the median expression [13].

### 2.3. Construction of Protein Interaction Network and Hub Gene Screening

The online STRING database (http://www.string-db.org/) was used to analyse and predict the DEGs and interactions between proteins encoded by these genes that may play an important role in the pathogenesis of IS. Medium confidence level (comprehensive score of interaction relationship between 0.4 and 0.7) was chosen as the significant standard. STRING analysis results were imported in Cytoscape 3.7.1 software for visualization. Briefly, CytoHubba and MCODE plug-in were used for protein-protein interaction network (PPIN) analysis. The degree at each node was calculated through CytoHubba. The network composed of the top 10 genes that ranked by the degree score can be regarded as the core module.

Three functional modules with the highest scores from PPIN screening by using molecular complex detection (MCODE) were also regarded as core subnetworks. Hub genes were identified by selecting the intersection of modules with the highest score and the top 10 genes of the highest degree scores and top 20 DEGs that met the above mentioned |Log2FC| criteria.

### 2.4. Hub Gene GO and KEGG Function Annotation

Biological functions (Gene Ontology, GO) of specific genes were analysed by using DAVID website (http://david.nciferf.gov). Kyoto Encyclopedia of Genes (Kyoto Encyclopedia of Genes and Genomes, KEGG, http://www.kegg.jp) was used for signal pathway analysis, which included cellular component (CC), molecular function (MF), biological process (BP), and KEGG pathways. Pathways are considered to be statistically enriched when *p* < 0.05.

### 2.5. Statistical Analysis

The R language glm function was used to construct the logistics model; the pROC package was used to perform receiver operating characteristic (ROC) curves on selected DEGs, where the area under the curve (AUC) of the hub genes were compared to identify the genes of significant prediction value. Finally, the ggplot2 package was used for ROC curve visualization.

## 3. Results

### 3.1. Differential Gene Expression

**Figure 1:**
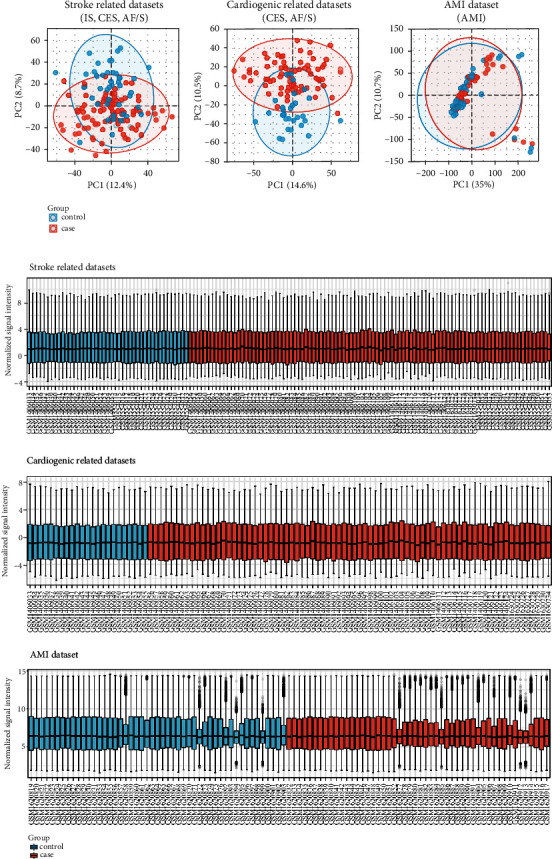
Validation and normalization of data. (a) Principal component analysis and (b) box plot of normalized data in the stroke-related datasets (GSE22255 and GSE58294 and GSE66724), cardiogenic-related datasets (GSE58294 and GSE66724), and AMI dataset (GSE66360).

Stroke-related datasets (GSE22255, GSE58294, and GSE66724) were merged, which included 43 normal control groups and 97 stroke groups. Cardiogenic-related stroke datasets (GSE58294 and GSE66724) were also merged, which include 23 normal control groups and 77 CES groups. Sample normalization was performed for each merged dataset, respectively. The normalization results showed that the median of each sample was basically on a horizontal line, which indicated a high level of normalization between samples (Figure 1(b)).

**Figure 2:**
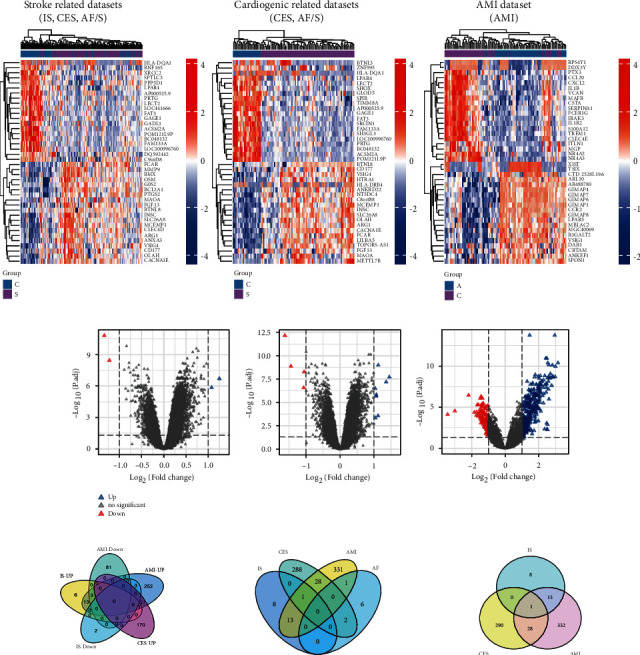
Heat maps (a) and volcano plots (b) showing DEGs for stroke-related datasets (GSE22255 and GSE58294 and GSE66724), cardiogenic-related datasets (GSE58294 and GSE66724), and AMI dataset (GSE66360). Venn plots (c) show the intersecting genes between different datasets.

Heat maps and volcano maps of stroke-related, cardiogenic-related, and AMI gene expressions are shown in Figures 2(a) and 2(b), respectively. After homogenization, there were 4, 236, and 460 DEGs that met the ∣log2(FC) | >1 and *p*.adj < 0.05 criteria. For stroke-related datasets (GSE22255, GSE58294, and GSE66724), there were 2 upregulation (positive log2FC) and 2 downregulation (negative log2FC) DEGs. For cardiogenic-related datasets (GSE58294 and GSE66724), there were 145 upregulation (positive log2FC) and 91 downregulation (negative log2FC) DEGs. For AMI dataset (GSE66360), there were 336 upregulation (positive log2FC) and 124 downregulation (negative log2FC) DEGs.

### 3.2. Intersecting DEGs

Based on the results of online Wayne analysis (Figure 2(c)), there were 13 genes that were jointly upregulated in IS and AMI diseases. There were 28 genes that were upregulated in CES and AMI diseases. There were 2 genes that were upregulated in CSE and AF diseases. There was 1 gene that was upregulated in AMI and AF diseases. Only one gene was upregulated in AMI, IS, and CES.

### 3.3. Tissue-Specific Gene Expression

34 genes were identified by using the online resource BioGPS (http://biogps.org) to study the tissue-specific expressions. Gene expressions with the highest tissue specificity was CD33+ myeloid (21.2%, 7/33) and smooth muscle (15.2%, 5/33). Besides, similar expression in cardiac myocytes and in whole blood (6.06%, 2/33) (Table 1) was found.

### 3.4. GO and KEGG Enrichment Analysis of DEGs

**Figure 3:**
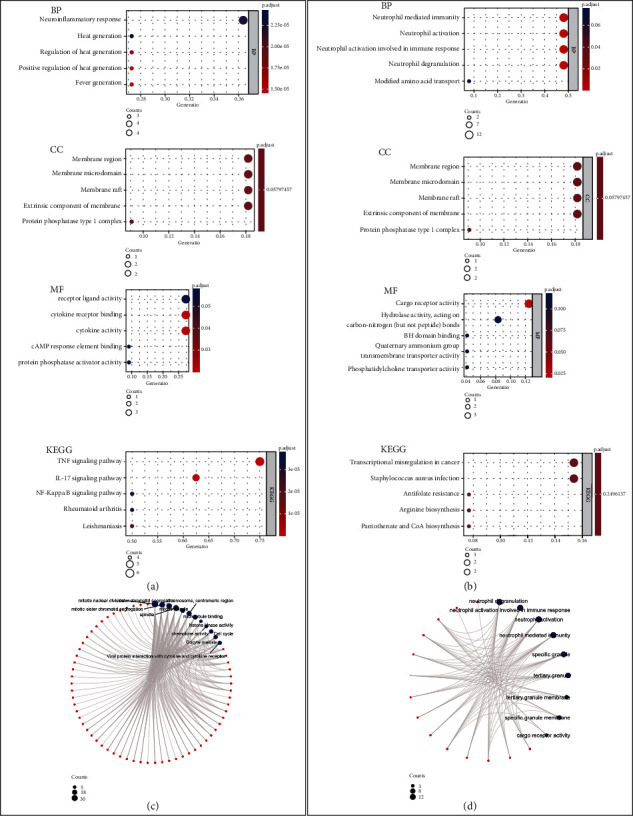
GO, KEGG analysis, and pathway visualization for AMI-IS (a, c) and AMI-CES (b, d) groups, respectively.

The GO and KEGG pathway analysis of the common genes between AMI and IS (Figure 3(a)) showed that the biological process (BP) changes mainly included heat generation, fever generation, neuroinflammatory response, and regulation of heat generation. Cellular components (CC) mainly enriched in the cell membrane such as cell membrane microstructure domain, membrane raft, extracellular components, and type 1 protein phosphatase complex. In terms of molecular function (MF), the significant changes were found in the cytokine receptor binding and cytokine activity, cAMP response element binding, and protein phosphatase activator activity. KEGG changes were mainly enriched in several immune system diseases (e.g., leishmaniasis and rheumatoid arthritis) and immune-related pathways (e.g., TNF signalling pathway, interleukin (IL-17) signalling pathway, and NF-*κ*B signalling pathway).

GO and KEGG pathway analysis of the common genes between AMI and CES (Figure 3(b)) showed that BP changes mainly included neutrophil-mediated immunity, neutrophil activation, neutrophil activation involved in immune response, neutrophil degranulation, and modified amino acid transport. CC changes were mainly in specific granules, tertiary granules, tertiary granular membranes, specific granule membranes, and Ficolin-1-rich granules. Significant changes in MF were the cargo receptor activity and hydrolase activity, BH domain binding, quaternary ammonium group transmembrane transporter activity, and phosphatidylcholine transporter activity. Changes in KEGG were mainly enriched in transcriptional misregulation in cancer, Staphylococcus aureus infection, arginine, and pantothenate and CoA biosynthesis, which, however, showed no significant difference. Enriched pathways are visualized in Figures 3(c) and 3(d).

### 3.5. PPI Network Analysis and Hub Gene Selection

**Figure 4:**
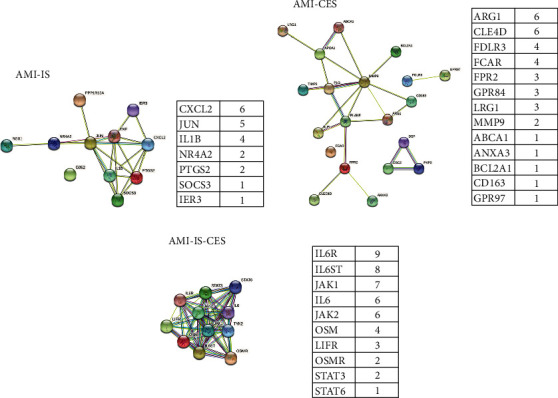
PPI for AMI-IS (a), AMI-CES (b), and AMI-IS-CES (c), respectively. Tables show the hub gene score in each disease group.

Cytoscape software was used to construct the PPI network to identify hub genes from common DEGs (Figure 4). The common hub genes in AMI and IS diseases (Figure 4(a)) are CXCL2 (C-X-C motif chemokine 2), which was produced by activated monocytes and neutrophils at the site of inflammation. In addition, hematopoietic regulatory chemokines that can inhibit the proliferation of hematopoietic progenitor cells in vitro increase hematopoietic cell activity. After the activation of the CAMP signalling pathway, JUN encodes the transcription factor AP-1, which promotes the steroid gene expression. IL1B (Interleukin-1 beta) is known as a potent proinflammatory cytokine. It was originally found to be the main endogenous pyrogen, which can induce the synthesis of prostaglandins, the influx and activation of neutrophils, the activation of T cells and the production of cytokines, the activation of B cells and the production of antibodies, and the proliferation of fibroblasts and the production of collagen and promote the differentiation of Th17 T cells.

The hub genes of CES and AMI (Figure 4(b)) were determined as ARG1 (Arginase-1), which is a key element of the urea cycle, and its role is to drive collagen synthesis and a bioenergy pathway that is essential for cell proliferation; CLEC4D (C-type lectin domain family 4 member D) acts as an endocytic receptor, which participates in the uptake of antigen at the site of infection or eliminates the antigen and presents it to T cells. FOLR3 (Folate receptor gamma) can bind to folic acid and reduce folic acid derivatives and mediate the delivery of 5-methyltetrahydrofolate to cells. FCAR (Immunoglobulin alpha Fc receptor) binds to the Fc region of immunoglobulin.

The hub genes screened for interaction between AMI and CES and IS diseases (Figure 4(c)) were IL6R (Interleukin-6 receptor) and IL6ST (Interleukin-6 receptor subunit beta), which are combined to activate and participate in the immune response, acute phase response, and regulation of hematopoietic functions. JAK1 (Tyrosine-protein kinase JAK1) participates in the IFN-*γ* signalling pathway and is the kinase chaperone of interleukin IL-2R receptor. IL6 (Interleukin-6) has various biological functions as a cytokine, and it is effective regulator for acute phase response. It plays an important role in the final differentiation of B cells into immunoglobulin secreting cells involved in the differentiation of lymphocytes and monocytes. It can act on B cells, T cells, hepatocytes, hematopoietic progenitor cells, and cells of the central nervous system and is essential for the production of TH17 cell. JAK2 (Tyrosine-protein kinase JAK2) nonreceptor tyrosine kinase is involved in various processes, such as cell growth, development, differentiation, or histone modification, which mediates the basic signal events in innate immunity and adaptive immunity. OSM (Oncostatin-M) is a growth regulator, which inhibits the proliferation of some tumor cell lines and regulates the cytokine production of endothelial cells.

### 3.6. Verification of Diagnostic Value of Hub Genes

**Figure 5:**
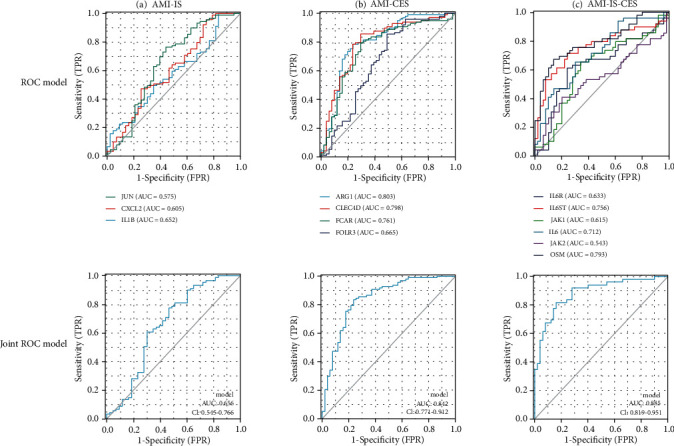
ROC and joint ROC model plots for AMI-IS (a), AMI-CES (b), and AMI-IS-CES (c) datasets.

In order to verify the diagnostic values of the first 6 hub genes obtained from the above analysis, the ROC curves constructed and the corresponding area under the curve (AUC) for the gene expression levels in the IS, CES, and AMI datasets were calculated. The first six central genes were diagnosed on ROC curves for AMI, IS, and CES diseases (Figure 5). In predicting normal and disease outcomes, JUN has a low prediction accuracy (AUC = 0.575, CI = 0.471 − 0.678), and CXCL2 has a low prediction accuracy (AUC = 0.605, CI = 0.498 − 0.712). IL1B has low prediction accuracy (AUC = 0.652, CI = 0.543 − 0.762). The combined model also showed low prediction accuracy (AUC = 0.656, CI = 0.545 − 0.766).

For AMI-CES datasets (Figure 5(b)), ARG1 showed certain level of accuracy in predicting normal and disease outcomes (AUC = 0.803, CI = 0.722 − 0.883), CLEC4D showed certain level of accuracy (AUC = 0.798, CI = 0.718 − 0.877), FCAR showed certain level of accuracy (AUC = 0.761, CI = 0.675 − 0.847), FOLR3 showed lower prediction accuracy (AUC = 0.665, CI = 0.564 − 0.765), and DeLong's test showed that ARG1 was superior to FOLR3 in predicting control and case outcomes (*p* = 0.015); CLEC4D was superior to FOLR3 (*p* = 0.016). The subsequent joint RCO model showed a certain level of accuracy (AUC = 0.842, CI = 0.771 − 0.912).

For AMI-IS-CES datasets (Figure 5(c)), OSM showed certain level of accuracy (AUC = 0.793, CI = 0.703 − 0.883), IL6ST showed certain level of accuracy (AUC = 0.756, CI = 0.656 − 0.855), IL6 showed certain level of accuracy (AUC = 0.712, CI = 0.611 − 0.814), JAK1 showed low prediction accuracy (AUC = 0.615, CI = 0.501 − 0.729), IL6R showed low prediction accuracy (AUC = 0.633, CI = 0.519 − 0.747), and JAK2 showed low prediction accuracy (AUC = 0.543, CI = 0.426 − 0.660). The subsequent joint RCO model, in predicting the outcome of control and case, showed certain level of accuracy (AUC = 0.885, CI = 0.819 − 0.951).

## 4. Discussion

Stroke is a cerebrovascular disease characterized by the sudden onset of symptoms. It is a common disease but involves high risks in the field of neurology. It has the characteristics of high morbidity, mortality, disability, and recurrence, which has serious negative impact on the patient's health and causes heavy burden to the stroke patients' family and society [13]. Cardiac stroke is a stroke type that occurs when a heart mural thrombus falls off due to various causes of heart disease and embolizes in the cerebral arterial system [14]. Stroke and myocardial ischemia are closely related, and both have a common pathophysiological basis: atherosclerosis [15]. There is a close relationship between the heart and the brain. With the progress of the disease, the risk gradually increases. Therefore, it is necessary to explore the molecular mechanisms of these two diseases to diagnose the early targets to prevent the development of the disease.

In this study, by searching IS, CES, AMI, and AF datasets from GEO, different datasets were combined, and common DEGs were screened out in IS, CES, AMI, and AF. GO enrichment and KEGG pathway enrichment analyses were performed, and PPI networks were established to identify the top hub genes from these common DEGs.

Human interleukin-6 (IL-6) gene is located at 7P21 [16], which is a type of glycoprotein with a relative molecular weight of 21~26 kDa. The main source of IL-6 in the blood is activated monocytes. When inflammation occurs in the body, monocytes and macrophages are the first reactive cells to express IL-6. When a stroke occurs, IL-6 in the brain is mainly derived from activated astrocytes and microglia [17, 18]. In addition, a variety of nucleated cells such as B cells, T cells, fibroblasts, and endothelial cells can secrete IL-6. IL-6 is a multifunctional inflammatory factor, which plays an important role in the inflammatory response by binding to the IL-6 receptor (IL-6R) on the tissue cells of the body [19].

Inflammation is an important pathological mechanism of AIS. When AIS occurs, the level of IL-6 in the patient's body increases rapidly in a short period of time, and the prognosis of AIS after 3 months is poor due to elevated level of inflammation, mainly in terms of the modified Rankin Scale (mRS), National Institute of Health Stroke Scale (NIHSS), and increased mortality. The higher the expression level of IL-6, the greater the risk of stroke recurrence [20]. In addition, studies have found that AIS can cause varying degrees of myocardial damage and arrhythmia [21, 22]. Within the three months of the occurrence of AIS, about 19% of patients have fatal or severe nonfatal heart disease [23]. Studies have shown that inflammatory response plays an important role in mediating cardiac function damage after stroke, which is characterized by excessive infiltration of inflammatory cells and the release of inflammatory mediators [24, 25].

The mechanism of cardiac function damage after the occurrence of AIS may be as follows: (1) IL-6 is an important inflammatory factor in the in vivo inflammatory response of atherosclerosis-related diseases. The larger the atherosclerotic plaque, the higher serum IL-6 level is found [26]. According to the TOAST classification, the most common type of ischemic stroke is aortic atherosclerosis, whose main pathological mechanism is the rupture of atherosclerotic plaque, leading to the occurrence of AIS. In atherosclerosis, the stability of the internal fibrous cap structure of atherosclerotic plaque is an important factor in determining whether the plaque can fall off. Collagen fibers are the skeleton of the fibrous cap matrix, and its content is regulated by the synthesis and degradation of collagen. Elevated IL-6 can stimulate the release of a variety of matrix metalloproteinases [27, 28], whose main function is to degrade collagen. After AIS, IL-6 levels in serum and cerebrospinal fluid increase rapidly in a short period of time, and the expression level of IL-6 is still rising after 1 week post AIS [29]. Increased IL-6 can accelerate collagen metabolism and cause atherosclerosis. The stability of the plaque is decreased, and the plaque is easy to fall off, which further contribute to the poor prognosis and the high recurrence rate of stroke. Atherosclerosis is also the main cause of cardiovascular disease. Studies have shown that increased IL-6 also increases the instability of cardiovascular atherosclerotic plaques, leading to angina pectoris, myocardial infarction, and other cardiac function impairments [30, 31]. (2) When AIS occurs, many microbubbles are released from the endothelial cells of the cerebral blood vessels into the circulation in the body, which could increase the level of IL-6 in the body [32]. Studies have confirmed that elevated level of IL-6, in addition to the increased instability of atherosclerotic plaques, can also cause vasospasm [33], thereby inducing the occurrence of coronary artery syndrome and leading to cardiac function damage [34]. Moreover, studies on IL-6 gene polymorphism have shown that IL-6 gene polymorphism is related to cardiovascular disease [35].

In this article, we merged the three datasets of AIS, CES, and AMI and found that IL-6 was one of the hub genes obtained by constructing PPI networks. It is proposed that IL-6 plays an important role in the three diseases. Therefore, after the occurrence of AIS, it is necessary to closely monitor the changes of IL-6 expression. If timely intervention and effective regulation of IL-6 expression in the body can be achieved, it can not only reduce the adverse prognosis of stroke but could also reduce the damage of heart function.

IL1B (Interleukin-1 beta) is located on chromosome 2 and is thought as a potent proinflammatory cytokine. It can exert biological effects through MAPK signalling, EGF-EGFR-RAS-JNK signalling pathway, IL1-IL1R-JNK signalling pathway, IL1-IL1R-p38 signalling pathway, and RAC/CDC42-PAK-ERK signalling pathway. It is produced extracellularly by the activation of Ras-MEKK1-JNK and MYD88-TRAF6-TAK1-p38-MAHKHK under the stimulation of cytotoxic drugs, pyrogens, reactive oxygen species, etc., which can induce the synthesis of prostaglandins, the influx and activation of neutrophils, T cell activation and cytokine production, B cell activation and antibody production, and fibroblast proliferation and collagen production and promote the differentiation of Th17T cells [36, 37]. It can also affect endothelial cells, including induction of adhesion factors and prothrombosis. In addition, if the balance of IL1B is disturbed, it can also lead to the development of atherosclerosis [38]. Studies have shown that local damage to the central nervous system can induce the activation of blood-derived inflammatory cells, which recruit the activated immune cells at the stroke site and produce and release proinflammatory and chemokines. Activated inflammatory cells will infiltrate the ischemic brain and aggravate the inflammation of neurons, leading to neuronal death [38]. Studies have shown that IL1B polymorphism is associated with IS genetic risk [39]. IL1B induces the gene expression of tissue factor and plasmin activator inhibitor I [40] and regulates the expression of multiple cell types to induce complex biological effects. The genetic variation of IL1B with higher inflammatory phenotypes can change Lp(a) in the risk of mediating long-term cardiovascular events [41]. These results indicate that IL1B can change many pathways involved in the development of atherosclerosis, thereby changing cardiovascular events.

Chemokines are small chemotactic cytokines that play an important role in regulating cell migration and can recruit white blood cells to the site of inflammation. CXCL2 is the ligand of CCR2, which is produced by activated monocytes and neutrophils, and is expressed in inflammation sites to induce macrophage infiltration. Activated macrophages can produce TNF-*α* and IL1B to promote neuronal cell death [42], and CXCR2 has a dual role after brain injury: (i) downregulation of homologous neurons CXCR2 will make neurons more susceptible to injury and (ii) chemotaxis and subsequent differentiation of blood-derived cells into a microglia-like phenotype, where the same receptor will be promoted [43]. Following the central nervous system injury, the rapid increase of CXCL2 will promote the transient neuronal CXCR2 downregulation, making neurons more susceptible to injury [44–46]. Chemokine receptors represent a promising target for reducing inflammation and secondary damage after brain injury. Georgakis et al. [47] conducted a genome-wide association analysis and found that higher CXCL2 has a higher genetic predisposition for IS and believed that the regulation of inflammatory response has great significance for the treatment of cerebral ischemia-reperfusion injury. Lee et al. [48] conducted animal experiments and found that intravenous injection of overexpressing CCL2-MSC (mesenchymal stem cells) showed increased angiogenesis and endogenous neurogenesis and reduced neuroinflammation [49]; hUC-overexpressing CCL2-MSCs have better functional recovery, which is due to the increased migration of these cells to brain regions where CCR2 expression is higher, which promotes the subsequent endogenous brain repair [50].

JUN (AP-1 transcription factor subunit) gene is involved in cell growth, development, and differentiation under normal conditions, but with very low expression level. Via the intracellular CRHR-PKA-ACTH signalling pathway, it can overactivate hippocampal neurons and cause cell death. Studies have suggested that JUN can be used as a marker for the progression of cell damage [51]. After IS, cells are stimulated to increase the number of positive cells expressing JUN gene [52, 53], neurotransmitter, and membrane depolarization, through LHCGR-GNAS-PKA signalling pathway that stimulates the second messenger to regulate key physiological processes, including metabolism, secretion, calcium balance, muscle contraction, cell fate, and gene transcription. cAMP acts directly on protein kinase A (PKA), exchange proteins activated by cAMP (Epac), and circulating nucleotide gated ion channels (CNGCs). PKA regulates some cell substrates through phosphorylation, including transcription factors, ion channels, transporters, exchangers, intracellular Ca2+ processing proteins, and contraction machinery, and plays a role in Ca+-No-cGMP, which causes Ca+ influx, increasing self-use radicals, and changes in NO, SOD, and adenosine which cause hypoxic brain damage.

ARG1 is a cellular enzyme mainly expressed in liver cells and a component of the urea cycle. It is also expressed in peripheral blood immune cells and participates in the immune response after the body is injured [54, 55]. It can exert anti-inflammatory effects by consuming L-arginine, inhibit the activity of TH1 cells, and enhance the proliferation ability of TH2 cells [56, 57]. Studies have shown that ARG1 is a direct target gene of miR-30a-5p, and the overexpression of miR-30a-5p can downregulate the expression of ARG1 protein in human neutrophils and participate in the pathophysiological process of IS. In patients with acute IS, ARG1mRNA continues to be upregulated and miR-30a-5p expression is downregulated [58]. Wang found that exosomes miR-30a-5p had lower expression level at the acute phase of IS. In addition, stroke patients with increased ARG1 are more likely to be infected and have a poor prognosis, which may be related to the immune suppression caused by the stroke. Previous studies [59, 60] suggest that miR-30a-5p and the target gene ARG1 are one of the important molecular markers of acute IS.

OSM (Oncostatin-M) belongs to the IL-6 family of cytokines. It is produced by activated macrophages and T cells. It can inhibit the growth of tumor cells, can induce the differentiation of certain tumor cells, and can also play a role in a variety of central nervous system diseases. It has the functions of inhibiting inflammation and exciting nerve damage, protecting neurons, and promoting the elongation of axons [61]. As a cytokine, OSM exists in the JAK/STAT signalling pathway. In mammals, the JAK/STAT pathway is the main signalling mechanism of a series of cytokines and growth factors. As cytokines bind to their cognate receptors, STATs are activated by members of the JAK family of tyrosine kinases. Once activated, they dimerize and translocate to the nucleus and regulate the expression of target genes to participate in innate and adaptive inflammatory host defence, cell growth, differentiation, cell death, angiogenesis, and development and repair processes aimed at restoring homeostasis. OSM can regulate the balance maintenance of astrocytes and microglia in the context of inflammation and neuroprotection [62]. It can increase the cellular activity of oligodendrocytes in the demyelinating site [63–65]. Studies have shown that OSM expression is increased in ischemic brain tissue, and OSM can increase endogenous SDF-1 expression in the brain by regulating STAT3 and ERK signalling pathways. In addition, OSM can reduce the expression level of IL-1*β* [66] while increasing the expression of IL-6 in astrocytes [67] and human brain endothelial cells [68]. IL-6 increases blood vessel endothelial permeability, increases the activity of coagulation factors, promotes coagulation, and causes vascular dysfunction. High concentration of IL-6 activates the expression of neutrophils and white blood cells, aggravates and maintains the degree of inflammation, damages vascular endothelial cells, and therefore induces minor cerebral haemorrhage [69–71]. The combined treatment of OSM and BMSCs in rats with IS can significantly improve the recovery of neurological function, reduce the area of brain damage [66], and increase the expression of nutritional factors.

CLEC4D (C-type lectin domain family 4 member D) is a C-type lectin receptor “Dectin-2 cluster,” which is mainly expressed by peripheral blood neutrophils, monocytes, and macrophages, and can transduce signal via SYk kinase of myeloid cells. After ligand binding, CLR stimulates the intracellular signal cascade to induce the production of inflammatory cytokines IL-6, IL-23, IL-12, and IL-1*β*, thereby triggering the innate adaptive immunity of pathogens. The innate immunity of CLEC4D is manifested in the expression on circulating neutrophils [72, 73]. CLECSF8 can also induce phagocytosis, proinflammatory cytokine production, and respiratory burst [74], which plays a role in pathogen recognition and clearance.

For the three hub genes selected from AMI and IS, the diagnostic value of IS combined with AMI was further investigated. The prediction AUC of JUN, CXCL2, and IL1B for IS and AMI were 0.575, 0.605, and 0.652, respectively. The prediction AUC of the joint model was 0.656. For the diagnosis of CSE and AMI, 4 hub genes were selected for disease diagnosis value prediction. The prediction AUC of ARG1, CLEC4D, and FCAR were 0.803, 0.798, and 0.761, respectively. The predictive power AUC of FOLR3 was 0.665. The prediction of the joint model has relatively high accuracy (AUC = 0.842). Further, DeLong's test for the above indicators showed that the diagnostic efficiency of ARG1 is better than that of FOLR3 and the diagnostic efficiency of CLEC4D is better than that of FOLR3. Validation of common genes for the three diseases shows that OSM has the highest prediction value, followed by IL6ST and IL6, JAK1, and JAK2. The joint model has high prediction accuracy (AUC = 0.918, CI = 0.867 − 0.970) in predicting the outcome of controls and cases. The above results show that a single gene has a low predictive ability for diseases, and when the hub genes are jointly used, its predictive ability is greatly improved. It also shows that when a disease occurs, it is not due to a single gene, but a network system built by multiple genes. The onset regulation function affects the occurrence, development, and outcome of the disease.

## 5. Conclusions

This work focuses on the datasets of IS, CES, and CES combined with AF and AMI. Since the dataset of CES combined with AF has less data, following differential expression analysis, no intersection with the other three datasets was found. Therefore, datasets that have intersections were further investigated, and it was concluded that the prediction accuracy of OSM gene is high, while OSM, IL6ST, and IL6 genes have better joint predictions in the above three diseases. Joint diagnosis using these 3 genes can further improve the prediction performance. However, this study still has certain limitations. This article uses bioinformatic methods to mine the GEO database that can predict the risk of IS, CSE, and AMI. The conclusions drawn are only based on bioinformatic mining, and further experiments or clinical studies are needed to confirm the prediction value.

## Figures and Tables

**Table 1:** Tissue-specific gene expression.

Gene	Name	System
NR4A2	Nuclear receptor subfamily 4 group a member 2	Adrenal cortex
CXCL2	C-X-C motif chemokine ligand 2	Smooth muscles/cd33^+^ myeloid
IL1B	Interleukin 1 beta	Smooth muscles
IER3	Immediate early response 3	Colorectal adenocarcinoma/bronchial epithelial cell/muscle/cardiac myocytes
PPP1R15A	Protein phosphatase 1 regulatory subunit 15a	cd33^+^ myeloid
RGS1	Regulator of g protein signalling 1	Olfactory bulb/bdca4+dentritic cells
JUN	Jun proto-oncogene, ap-1 transcription factor subunit	Lung
SOCS3	Suppressor of cytokine signalling 3	Skeletal muscle
PTGS2	Prostaglandin-endoperoxide synthase 2	Smooth muscles
G0S2	G0/g1 switch 2	Adipocyte
TNF	Tumor necrosis factor	721 b lymphoblasts
CLEC4D	C-type lectin domain family 4 member d	cd33^+^ myeloid
ANXA3	Annexin a3	Bronchial epithelial cell
VNN3	Vanin 3	cd33^+^ myeloid
TNFAIP6	Tnf alpha-induced protein 6	Smooth muscles
BCL2A1	Bcl2-related protein a1	Whole blood
CD163	Cd163 molecule	Cardiac myocytes
FOLR3	Folate receptor gamma	cd14^+^ monocytes
FPR2	Formyl peptide receptor 2	Whole blood
WDFY3	Wd repeat and fyve domain containing 3	Thalamus
FCAR	Fc fragment of iga receptor	cd33^+^ myeloid
LRG1	Leucine-rich alpha-2-glycoprotein 1	Liver
MMP9	Matrix metallopeptidase 9	Bone marrow
RNASE1	Ribonuclease a family member 1, pancreatic	Testis Leydig cell
ADGRG3	Adhesion g protein-coupled receptor g3	Whole blood
MCEMP1	Mast cell expressed membrane protein 1	cd33^+^ myeloid
DSC2	Desmocollin 2	Colon
SLPI	Secretory leukocyte peptidase inhibitor	Trachea
SLC22A4	Solute carrier family 22 member 4	cd71^+^ early erythroid
ABCA1	Atp binding cassette subfamily a member 1	Smooth muscles
FBN2	Fibrillin 2	Placenta
ARG1	Arginase 1	Fetal liver
PLXDC2	Plexin domain containing 2	cd33^+^ myeloid
OSM	Oncostatin M	cd71^+^ early erythroid

## Data Availability

The data used to support the findings of this study are included within the article.
